# Chronic Mania: Diagnostic Dilemma and the Need for Addition in Nosology

**DOI:** 10.7759/cureus.38703

**Published:** 2023-05-08

**Authors:** Surbhi Batra, Abhinav Anand, Anmol Singh, Shrestha Verma

**Affiliations:** 1 Psychiatry, Atal Bihari Vajpayee Institute of Medical Sciences and Dr. Ram Manohar Lohia Hospital, New Delhi, IND; 2 Psychiatry, All India Institute of Medical Sciences, Jodhpur, Jodhpur, IND; 3 Covid Care, Central Jail Hospital, Mandoli Prison, New Delhi, IND; 4 Psychiatry, Medanta Institute of Neurosciences, Gurugram, IND; 5 Psychiatry, St. Stephen’s Hospital, New Delhi, IND

**Keywords:** mania, nosology, bipolar disorders, mood stabilizer, chronic mania

## Abstract

Chronic mania is a mental health disorder that has been described by various psychiatrists in the past but currently is not a part of nosology. Robust epidemiological data for chronic mania are lacking with regard to its prevalence and clinical features.

The present case report is of a 48-year-old male with a six-year history of mood and psychotic symptoms, based on which differential diagnoses of schizoaffective disorder (manic type), schizophrenia, and mania with psychotic symptoms (with chronic course) were made. The diagnosis of chronic mania was confirmed considering the predominance of fluctuating mood symptoms along with psychotic symptoms, lack of remission, and chronic course of illness. Antipsychotics were initially started for six weeks, to which the patient demonstrated a minimal response. A mood stabilizer was added to the regimen, leading to significant improvement, and the patient was discharged.

According to existing literature, patients with chronic mania present with severe illness, the presence of psychotic symptoms, and socio-occupational impairment, which was also noticed in this case. The prevalence of chronic mania among patients with bipolar disorder is approximately 13-15%, which constitutes a significant proportion of known mental illnesses. Therefore, chronic mania should be added as a distinct clinical entity in the existing nosological systems.

## Introduction

Chronic mania is characterized by the presence of manic signs and symptoms for more than two years without remission [1]. Recent literature defines chronic mania as a lack of at least two points of improvement from baseline on the Clinical Global Impression-Bipolar Disorder Mania (CGI-BP) scale at any point of observation during the 12 months after the initial treatment for acute mania [2]. Chronic mania is often difficult to diagnose because of overlapping cyclothymic or hyperthymic temperament; symptoms superimposed between mania, schizophrenia, and schizoaffective disorders [3]; or concurrent cannabis use [4]. In India, a chronic and recurrent manic pattern has been seen to be more prevalent than typical bipolarity [5,6]. The current study is a case of mania presenting as the first episode with a diagnostic dilemma and typical clinical features of chronic mania. In this case, we propose chronic mania be added to the nosological classification system and discuss the rationale behind it.

## Case presentation

Mr. A, a 48-year-old unmarried male, educated up to class 10th and unemployed since 2014, with a well-adjusted premorbid personality, belonging to a Hindu nuclear family of lower socioeconomic status and no significant family history of psychiatric illness, presented with manic symptoms for a total duration of six years (2014), with acute onset and continuous course characterized by irritability, over-talkativeness, over-religiosity, overplanning, overfamiliarity, authoritativeness, decreased need for sleep, increased appetite, and delusion of grandiosity followed by ideas of persecution. Initially, when the patient started having symptoms in 2014, he was predominantly irritable, overtalkative, overgrooming, boastful of himself and his abilities, and had an increased appetite and a decreased need for sleep. As opposed to his usual sleep of around seven hours at night and one hour of sleep in the afternoon before the onset of illness, the patient stopped sleeping in the afternoon. Gradually, over the next couple of weeks, he also started sleeping less at night which eventually reduced to two hours only. Despite the significantly decreased duration of sleep, he was noticed to be more energetic during the day as well as at night. He would engage in multiple activities and roam about inside the house or in the neighborhood.

After three to four months, family members realized that he was making unrealistic claims about himself such as being a spiritual leader. He would groom himself accordingly, wearing vermillion and multiple religious necklaces. He claimed that he could do anything and would bless children to become doctors and lawyers and firmly believed that his blessing would become a reality. He also claimed to have blessed individuals who are currently famous actors and even the prime minister although he had never met them. After a year of the onset of illness, the patient also had persecutory ideas such as some secret organization will kidnap him to take advantage of his talents. Additionally, his appetite and self-care reduced compared to his overgrooming behavior before. Even though these symptoms would improve for one to five days occasionally in the course of the next five years, they largely remained constant with no functional improvement noted by the family. All of these symptoms were also associated with increased psychomotor activity and aggressive behavior. Apart from these symptoms, the patient was consuming 1-1.5 packs of cigarettes/day for the past 20 years. The patient was taken to multiple faith healers from 2014 to 2019 as the family believed that the illness was due to supernatural powers. The patient had never received any medical treatment until June 2019 which further contributed to his significant socio-occupational decline. The patient received his first session of treatment in 2019 for two months from June to July 2019 using oral tablets and injections with an improvement in his duration of sleep only. The exact details of medications were not available. The patient left the treatment owing to the financial burdens posed by the treatment.

The patient was admitted to the psychiatry ward in June 2020 and initially required injectables to control agitative behavior. There was no past history of mental illness, similar episodes, or substance use in the patient. Early developmental history revealed no abnormality, and his premorbid personality lacked any hyperthymic traits. There was no family history of psychiatric or neurological illness. There were no significant findings on physical examination. Routine investigations including thyroid function tests were normal, screening for drugs and toxins was normal, and electroencephalography and CT scans revealed no pathology. There was no history indicative of Schneider’s first-rank symptoms, head injury, or seizures.

Differential diagnoses of schizoaffective disorder (manic type), schizophrenia, and mania (having chronic course) with psychotic symptoms were considered. During further detailed evaluation and observation of the patient’s behavior in the ward, it was found that the illness began with a fluctuating mood in the form of irritability and anger outbursts with other symptoms such as a decreased need for sleep and increased appetite. Symptoms such as delusion of grandiosity and delusion of persecution appeared only after vegetative and mood symptoms. The diagnosis of schizophrenia was excluded considering the prominence of mood symptoms throughout the course and the absence of hallucinatory behavior and negative symptoms. On further clarification of history, it was found that the mood symptoms and the psychotic symptoms had been present together at the same time throughout the illness.

As per the International Classification of Diseases 11th Revision (ICD-11), schizoaffective disorder is “an episodic disorder in which the diagnostic requirements of schizophrenia and a manic, mixed or moderate or severe depressive episode are met within the same episode of illness, either simultaneously or within a few days of each other” [7]. However, the absence of typical schizophrenic symptoms and lack of episodic course of illness ruled out this diagnosis per ICD-11. According to the Diagnostic and Statistical Manual, fifth edition (DSM-5), for the diagnosis of schizoaffective disorder, “delusions or hallucinations should be present for two or more weeks in the absence of major mood episode during the lifetime duration of the illness” [8]. However, this criterion was not met in this patient, as the delusions were present concurrently with the persistent irritable mood throughout the course of the illness. Hence, the diagnosis of chronic mania was made considering the predominance of mood symptoms along with psychotic symptoms, lack of remission, and chronic course of illness.

The patient was initially admitted and evaluated based on the Young Mania Rating Scale (YMRS) with a baseline score of 51 and severely ill (6) on the CGI-BP. He was administered olanzapine 10 mg per day and gradually built up to 20 mg per day by the second week. Parenteral administration of a combination of haloperidol and promethazine was given as needed for agitation. The patient required parenteral administration of antipsychotics every day till week three and only showed mild improvement in biological functions such as sleep by week four, and YMRS still showed a score of 48. Therefore, valproate was added to the treatment and gradually built up to 1,500 mg/day until serum levels of 110.0 mg/dL were obtained by week six. YMRS score at week six reduced to 32 and he no longer needed parenteral antipsychotics. The patient was discharged from the ward at the end of week eight on olanzapine 20 mg/day and valproate 1,500 mg/day with a YMRS score of 24 with moderately ill (4) on the CGI-BP.

**Figure 1:**
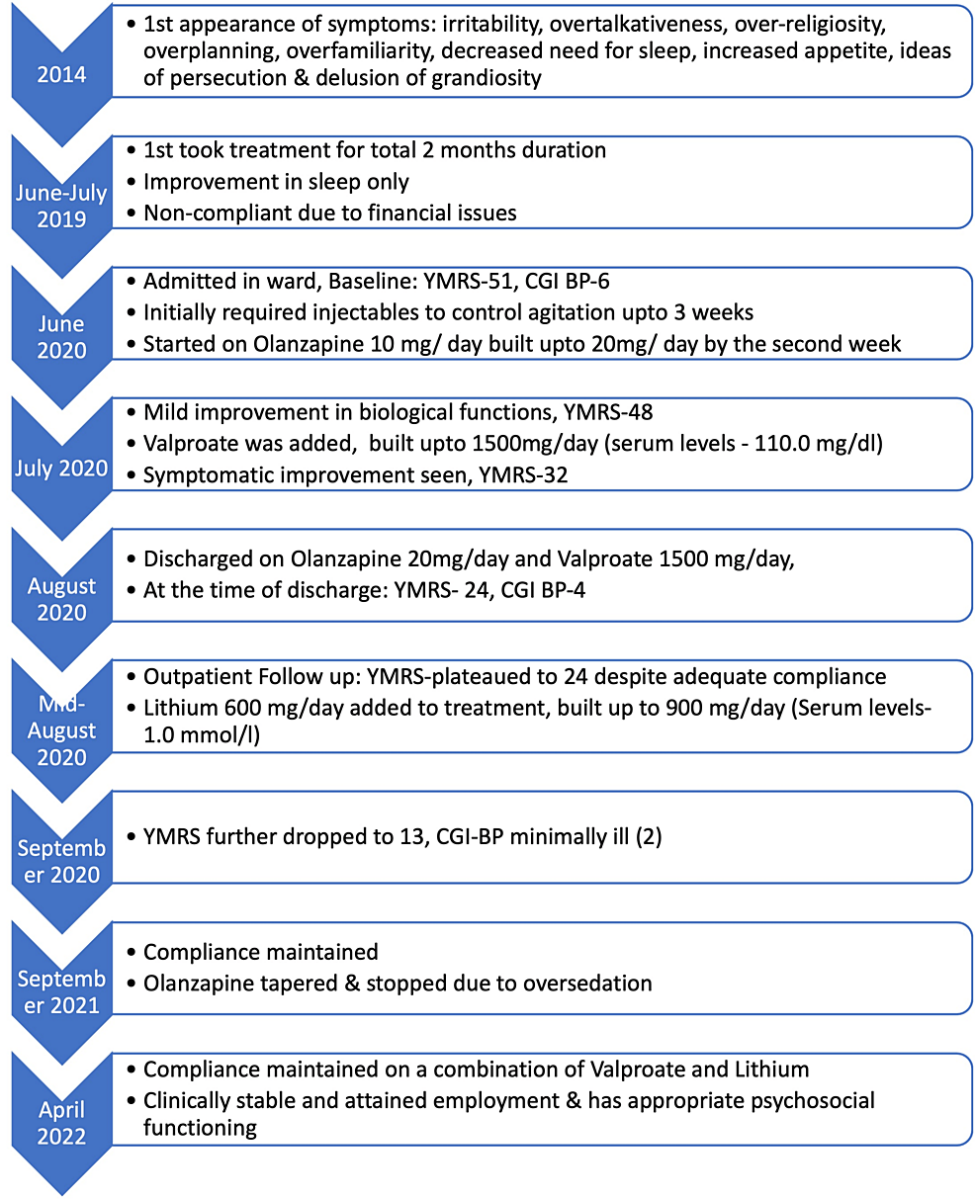
Timeline of key events. YMRS: Young Mania Rating Scale; CGI-BP: Clinical Global Impression Bipolar Severity Scale

Following discharge, the patient was followed up on an outpatient basis, and it was seen that his YMRS score had plateaued to 24 for the next two weeks despite maintaining valproate levels at 112 mg/dL. Hence, it was decided to add lithium to the management regime. Lithium 600 mg/day was started at week 10 after pre-lithium workup and built up to 900 mg/day with blood levels of 1.0 mmol/L. Upon the addition of lithium, the YMRS score further dropped to 13 at week 14 with much minimally ill (2) on Clinical Global Impressions of Bipolar Severity. After one year of follow-up, because of oversedation, olanzapine was tapered and stopped. The patient maintained compliance and was stable on a combination of valproate and lithium and has been following up regularly since then. He even got employed and has appropriate psychosocial functioning. A brief timeline of key events is shown in Figure 1.

## Discussion

In its entirety or compared to its depression equivalent (dysthymia), chronic mania has been a grossly understudied and underdescribed entity. Although chronic mania does not hold nosological status in the current classification systems, i.e., ICD-11 and DSM-5, it has been described in the literature by many clinicians from the 19th and 20th centuries, such as Pinel (1802), Esquirol (1838), Griesinger (1865), Schott (1904), Kraepelin (1913), and Wertham (1929) [9].

Chronic mania has been described as the persistence of manic symptoms for more than two years without remission. In the current case, it lasted for roughly six years without remission [10,11]. Robust epidemiological studies are lacking with regard to the prevalence and clinical features of chronic mania. Most studies on patients with bipolar and related disorders show a proportion of 6-15% for chronic mania [2,10-13]. European Mania in Bipolar Longitudinal Evaluation of Medication (EMBLEM), a European Cohort study, found that 15% of patients with bipolar and related disorders met the criteria of chronic mania. Chronic mania was found to be associated with relatively less severe manic symptoms, less socially active, and greater occupational impairment [2]. Regarding clinical presentation, past literature, such as by Schott (1904), described it as a milder presentation of acute mania, with minor loss of mental activity, memory, attention, and knowledge. In addition, a marked loss in judgment and insight was observed [9]. Later, studies differentiated chronic mania from acute mania as having a high rate of almost constant euphoria, grandiose delusions, and related delusions, as well as relatively low rates of sleep disturbance, psychomotor agitation, and hypersexuality [2]. Chronic mania is underrepresented in the clinical population probably due to superimposed hyperthymic temperament or overlap of symptoms of schizophrenia or schizoaffective disorders [2].

According to existing literature, manic episodes usually resolve without treatment within four to six months [14]. The index case is unique in the presentation as the symptoms of the illness were reported for the first time in the form of mania lasting for six years without remission and without a case history or family history of mental illness or substance use apart from tobacco. Most cases reported in the literature usually have either of those [1,2,4,9,11,13].

Commonly, chronic mania is characterized by a lack of features such as reduced sleep, agitation, the pressure of speech, and hypersexuality but is reported with the presence of euphoric, grandiose, and psychotic features [11]. However, the index case presented with a lack of hypersexuality and had grandiose delusions, reduced sleep, and agitation. Management of the condition involves a step-wise introduction of a combination of mood stabilizers and neuroleptics [14]. Often chronic mania is difficult to treat; however, in this case, the patient was relatively responsive to drugs.

Patients with chronic mania exhibit severe illness, the presence of psychotic symptoms, and socio-occupational impairment [2,15], which is similar to the index case. The proportion of chronic mania is approximately 13-15% [2,11] and is considered to be relatively large and significant in the context of mental illnesses. Hence, it is highly recommended that chronic mania be added as a distinct clinical entity in the existing nosological systems.

## Conclusions

Bipolar and related disorders have a considerably significant proportion of patients with chronic mania. Often a misdiagnosed, underrecognized clinical entity, patients with chronic mania exhibit greater severity of illness, coexisting psychotic symptoms, and significant socio-occupational impairment. Despite being described extensively in the literature, it is not included in the existing nosological systems. This article describes a case of chronic mania to emphasize the need for its inclusion as a distinct clinical entity in nosology.
